# Cancer care interventions for forcibly displaced populations in low- and middle-income countries of the Middle East and North African region affected by humanitarian crises: Protocol for a scoping review

**DOI:** 10.1371/journal.pone.0327946

**Published:** 2025-08-18

**Authors:** Anne Lyon Havlik, Zena Ahmed, Philippa Susan Chadwick, Kate Nyhan, Kaveh Khoshnood

**Affiliations:** 1 Department of Epidemiology of Microbial Diseases, Yale School of Public Health, Yale University, New Haven, Connecticut, United States of America; 2 Harvey Cushing/ John Hay Whitney Medical Library, Yale University, New Haven, Connecticut, United States of America; 3 Department of Environmental Health Sciences, Yale School of Public Health, Yale University, New Haven, Connecticut, United States of America; Johns Hopkins Medicine, UNITED STATES OF AMERICA

## Abstract

**Objective:**

The objective of this scoping review is to describe and assess what cancer treatment interventions exist for forcibly displaced populations living with cancer in LMIC humanitarian settings in the Middle East and North African (MENA) region and what research has been done on these interventions.

**Background:**

Humanitarian aid usually provides short-term requirements for food, shelter, and trauma relief; long-term relief focuses mainly on communicable diseases and not non-communicable diseases (NCDs) like cancer. However, no systematic search and synthesis resource of existing cancer care interventions for forcibly displaced populations in LMICs of the MENA region exists.

**Inclusion criteria:**

Original studies will be included on interventions for all types of cancer which are available to support forcibly displaced populations in settings of humanitarian crisis in Djibouti, Egypt, Jordan, Lebanon, Mauritania, occupied Palestinian Territories, Somalia, Sudan, South Sudan, Syria, and Yemen.

**Methods:**

A scoping review will be conducted in accordance with JBI methods and guidelines and PRISMA reporting guidelines (**S3 Appendix**). PubMed, Global Health, Embase, Scopus, The Lens, Global Index Medicus, and CINAHL Complete will be searched for peer-reviewed articles published from January to until present. IOM, ReliefWeb, OSCE, UNHCR, WHO, OCHA, and ACLED will be searched for grey literature. A dual review using three reviewers will independently identify all relevant articles and extract data from them. A narrative summary of findings will be reported,

**Discussion:**

This scoping review will provide an overview of the available cancer interventions for forcibly displaced populations and describe the research done regarding these interventions. Potential gaps in the field will be identified,

**Systematic Review Registration:**

Zenodo, https://doi.org/10.5281/zenodo.14633092

## Introduction

Humanitarian crises across the world, such as major disease outbreaks, armed conflict, and natural disasters, affect more people today than at any other point in recorded human civilization [1]. These crises present both immense acute and long-term health impacts on hundreds of millions of people [2]. People living in low and middle-income countries (LMICs) disproportionately bear the health burden of humanitarian crises [3]. Individuals living in LMICs are generally impacted more frequently by these crises, but have less preparation for them, including in the healthcare delivery system [3]. Furthermore, oftentimes multiple types of humanitarian crises occur simultaneously, for example, famine or major disease outbreaks occurring with armed conflicts [3],

Much of the Middle East and North Africa (MENA) region has experienced protracted conflict affecting several of its regions over the past few decades. Multiple countries in the MENA region are actively involved in armed conflicts, which have distressed their already taxed economies and subsequently impacted the resources available to healthcare delivery [4]. Throughout the past decade, the number of people in humanitarian conflicts, including both the non-resolution of current conflicts and the uprising of new conflicts, has surged in the MENA region [5]. The increase in humanitarian crises occurs in LMIC settings with vulnerable healthcare systems, where coordinated responses are less likely due to economic and structural constraints [6]. Furthermore, humanitarian crises have disproportionately interrupted critical, ongoing care and prevention for people with non-communicable diseases (NCDs), like cancer [7],

As humanitarian emergencies cause disruption to the provision of health services for those with NCDs, responses to NCD care needs in these challenging emergency contexts lack an evidence base to inform the response [8]. Resultantly, people who have been forcibly displaced may experience interrupted cancer treatment or the development of new cancer. These populations often present with advanced disease and suffer more complications, compounded by issues such as poor hygiene and living conditions, limited access to care, and limited resources available to them [6]. With the growing global burden of cancer and the mass displacement of populations in the MENA region from humanitarian crises, forcibly displaced populations living with cancer represent a particularly neglected community in global health,

Humanitarian aid usually targets short-term needs for food, shelter, and trauma relief, focusing on communicable diseases, and not non-communicable diseases like cancer. Preparation for humanitarian crises is costly in LMICs, including building structure and support for emergency preparedness mechanisms, disaster risk reduction, and social protection to absorb systemic shocks from humanitarian crises [9]. Likewise, cancer care has been considered too costly and too complex, with a lack of cost-effective interventions as well as host health systems’ inability to expand cancer services and interventions [8]. Individuals who are already diagnosed with cancer face deficient healthcare facilities and resources, often forced to migrate to seek expensive cross-border therapy interventions, given the limited international aid available [10–13]. These settings and humanitarian aid must first reliably provide cancer care treatment and interventions prior to ethically providing screening, following the fundamental ethics principles put forth by Wilson and Jungner [14]. Therefore, we seek to describe the cancer interventions available in humanitarian settings. In this review, we will summarize and discuss the research available involving humanitarian crises with respect to the MENA region and provide recommendations for where humanitarian assistance can fundamentally fill in the gaps of cancer interventions, with a focus on building local healthcare system capacities and strengthening healthcare systems to improve health-related outcomes at all stages of cancer diseases,

A preliminary search of MEDLINE, the Cochrane Database of Systematic Reviews, and JBI Evidence Synthesis was conducted, and no current scoping reviews on cancer care interventions for forcibly displaced populations living with cancer in humanitarian settings of LMICs in the MENA region were identified,

A scoping review will be conducted to systematically map the interventions and research done in the area of cancer care interventions for forcibly displaced populations living with cancer in humanitarian settings of LMICs in the MENA region. The goal is to identify the types of interventions for cancer, aggregate information about what published research has been done on these interventions, and identify existing gaps in knowledge for cancer care interventions,

## Review question(s)

What cancer treatment intervention(s) exist for forcibly displaced people living with cancer in LMIC humanitarian settings in the MENA region? What research has been done on these interventions?

## Design

### Participants

Participants to be included in this scoping review are forcibly displaced persons living with any type of cancerous disease and any stage/grade of cancer (including both non-metastatic and metastatic) who reside in LMICs recognized as experiencing a humanitarian crisis in the MENA region at the time the study was conducted. There will be no restrictions on participants’ age or gender in this study.

### Inclusion criteria

This scoping review will consider published research (peer-reviewed and grey literature using primary data) investigating forcibly displaced populations living with cancer in LMICs of the MENA region. Material published in any language will be considered. The review team will evaluate and synthesize results from resources published in English and create an Appendix of resources identified as relevant but published in another language.

### Exclusion criteria

This scoping review will exclude commentaries, correspondences, conference abstracts, editorials, and opinion pieces from consideration. This scoping review will document studies from January 1999 onwards, reflecting the advancements in cancer interventions during that time frame.

### Concept

The concept of this project is to conduct a scoping research project that screens cancer interventions in humanitarian settings in LMICs of the MENA region. The screening process plays a crucial role in identifying the types of intervention strategies required for the treatment of cancerous diseases. By conducting a scoping review, this study provides a comprehensive understanding of the landscape of interventions and cancer care within the MENA region. Furthermore, this scoping review project serves as a valuable tool for recognizing the gaps of humanitarian settings that have a paucity of medical support.

### Context

This scoping review aims to explore and examine research conducted in low and lower-middle-income countries confronting humanitarian crises within the MENA region. The MENA region is defined according to the World Bank classification, which includes a diverse group of countries spanning both the Middle East and North Africa. This delineation broadly aligns with the World Health Organization Eastern Mediterranean Region (WHO EMR), although there are notable differences, such as the inclusion of countries like Israel and the exclusion of others such as Sudan, Somalia, and Pakistan. While the occupied Palestinian territory is not classified as a sovereign state within this grouping, it is included in this review due to the severity of its ongoing humanitarian crisis and its frequent inclusion in global health and humanitarian literature focusing on MENA settings [15].

Countries will be included or excluded based on their World Bank income level rating, obtained via the World Bank Open Data [16]. However, in the case of the occupied Palestinian territory, where the information from the World Bank Open Data is unavailable, the United Nations Children’s Fund (UNICEF) classification of a ‘lower-middle income’ economy is utilized [17]. The inclusion criteria encompass countries with a World Bank rating of Middle Income (excluding Upper Middle-Income ratings) or below. The countries with a rating less than “Upper” and “Middle-Income Countries,” Algeria, Comoros, Djibouti, Egypt, Jordan, Lebanon, Morocco, Mauritania, occupied Palestinian territory, Somalia, Sudan, South Sudan, Syria, Tunisia, and Yemen, are included.

Countries that have experienced humanitarian crises are selected based on their current humanitarian status via the United Nations Office for the Coordination of Humanitarian Affairs’ Reliefweb Database. In this database, Palestine is referred to as “occupied Palestinian territory” [18]. With respect to the current humanitarian crisis, these selected countries, within the scoping review, currently face an ongoing humanitarian situation: Djibouti, Egypt, Jordan, Lebanon, Mauritania, occupied Palestinian Territories, Somalia, Sudan, South Sudan, Syria, and Yemen.

Table 1 provides a detailed description of the list of countries mentioned above, encompassing The World Bank Group’s classification of fragile and conflict-affected situations from 2005 (World Bank documentation started in 2005) to 2023 [16,19]. The ongoing crises for each country are represented and specified according to their severity, utilizing the ACAPS database [20]. Additionally, Table 1 incorporates the Gross Domestic Product (GDP) of each listed country, solidifying the inclusion and exclusion criteria based on their GDPs. This integrated format facilitates an understanding of the dynamic interplay between a nation’s socio-political circumstances, economic indicators, and the severity of the crises.

**Table 1:** Country level overview for fragile and conflict-related conditions.

Country	Years Experiencing Fragile and Conflict-affected Situations 2005–2023 [19]		2024 Type of Ongoing Crisis^*^	2024 INFORM Index of most severe crisis^**^	GDP of country (World Bank data in current US$ millions)^***^
Djibouti	2006-2009 2017-2019	7 years	Hunger crisis and climate crisis (Drought)	2.8	3,136.1
Egypt	N/A	0 years	(Syria Regional Crisis)^****^	4.2	4,295.4
Jordan	N/A	0 years	Syria Regional Crisis	4.2	4,311.0
Lebanon	2016-2023	7 years	Syria Regional Crisis	4.2	4,136.1
Mauritania	2007	1 year	Food security	2.6	2,065.2
Occupied Palestinian Territories	2006-2023	17 years	Conflict	4.2	Not available
Somalia	2005-2023	18 years	Complex crisis (displacement, violence, floods, drought)	4.7	592.1
Sudan	2005-2023	18 years	Complex crisis (displacement, violence, floods)	4.8	1,102.1
South Sudan	2013-2023	10 years	Complex crisis (Conflict, floods, displacement)	4.5	1,071.8
Syria	2013-2023	10 years	Syrian Conflict	4.5	420.6
Yemen	2009-2023	14 years	Conflict in Yemen	4.7	650.3

* Humanitarian disasters result from armed conflicts, epidemics, famine, natural disasters, and other significant emergencies [1]. Natural disasters can be earthquakes, floods, storms, or disease outbreaks [1]. Man-made emergencies such as armed conflicts, plane and train crashes, and other industrial-related incidents [1]. Complex emergencies involving a mix of natural and human-made factors create vulnerability within the population, resulting in a humanitarian crisis [1].

** Source: [20]

*** Source: [16]

**** The Syrian regional crisis has regional implications for neighboring countries, amid the deteriorating socio-economic and humanitarian conditions of those countries [21].

### Types of sources

Studies selected must be interventional. This scoping review will consider both experimental and quasi-experimental study designs, including randomized controlled trials, non-randomized controlled trials, before-and-after studies, and interrupted time-series studies. In addition, analytical observational studies, including prospective and retrospective cohort studies, case-control studies, and analytical cross-sectional studies, will be considered for inclusion. This review will also consider descriptive observational study designs, including case series, individual case reports, and descriptive cross-sectional studies for inclusion.

Qualitative studies will also be considered that focus on qualitative analysis via narrative research, phenomenological research, grounded theory research, ethnographic research, and historical research. In addition, systematic reviews and scoping reviews that meet the inclusion criteria will be included.

## Methods

The proposed scoping review will be conducted in accordance with the Joanna Briggs Institute (JBI) methodology for scoping reviews [22]. The review protocol has been posted on Zenodo (10.5281/zenodo.14633092) and is being reported in accordance with the reporting guidance provided in the Preferred Reporting Items for Systematic Reviews and Meta-Analyses Protocols (PRISMA-P), S3 Appendix [23].

### Search strategy

The search strategy will aim to locate both peer-reviewed studies and non-peer-reviewed sources. The search will include literature published from January 1999 to November 19, 2024. The PubMed search strategy, found in the S1 Appendix, used Medical Subject Headings (MeSH), and text words contained in the titles and abstracts of relevant articles and was developed by the research team in collaboration with a health sciences librarian. This search string will be adapted to execute a similar search in Global Health (via Ovid), Embase (via Ovid), Scopus, The Lens, Global Index Medicus, and Cumulative Index to Nursing and Allied Health Literature (CINAHL) Complete (via Ebscohost). These database searches will be augmented by a gray literature search of the International Organisation for Migration, ReliefWeb, Organizations for Security and Co-operation in Europe (OSCE), United Nations High Commissioner for Refugees (UNHCR), World Health Organization (WHO), Office for the Coordination of Humanitarian Affairs (OCHA), and The Armed Conflict Location and Event Data Project (ACLED). The search for references will be run across all databases and gray literature sources. Zotero, a bibliographic software, will be used to store, organize, and manage all references. All retrieved sources will be screened.

### Study selection and data extraction

Following the searches of the selected databases and information sources, all identified bibliographic records will be uploaded into Covidence systematic review software. Duplicate bibliographic records will be removed. Following a pilot test, titles and abstracts will then be screened using the dual reviewer paradigm with three independent reviewers, having two votes per decision, for assessment against the inclusion criteria for the review in Covidence. Then, the selected citations will proceed to full text review and be assessed in detail against the inclusion criteria by three reviewers independently using a dual review paradigm. Reasons for the exclusion of sources of evidence that do not meet the inclusion criteria during full-text screening will be recorded and reported in the scoping review. Any disagreements that arise between the reviewers at each stage of the selection process will be resolved through discussion or with an additional reviewer. Data will be extracted from all of the selected papers that pass the full text review, pulling out the information in the extraction form. S2 Appendix contains a draft extraction form which will be modified and revised as necessary during the process of extracting data from each included evidence source. Modifications will be detailed in the scoping review. Conflicts between reviewers in the data extraction phase will be resolved through discussion or with an additional reviewer. The results of the search and the study inclusion process will be reported in full in the final scoping review and presented in a Preferred Reporting Items for Systematic Reviews and Meta-analyses-2020 flow diagram [24].

### Data Analysis

The information gathered from the included studies via the data extraction tool will be collated. This data will be shown in a table of major study characteristics and findings, along with an evidence gap map. Furthermore, results will be followed by a discussion contextualizing the results related to the research question: What cancer treatment intervention(s) exist for forcibly displaced populations living with cancer in LMICs humanitarian settings in the MENA region? What research has been done on these interventions?

Data analysis will be carried out through a team approach for the review process, data extraction, analysis, and presentation of findings [25]. The results from the data extraction tool developed (S2 Appendix) will be systematically reviewed, utilizing frequency counts to generate descriptive statistics that will inform conclusions for practice and policy recommendations. This will be followed by a discussion of the limitations of the scoping review process and the conclusion.

## Results and discussion

The results and dissemination of the findings will identify the gaps in cancer care and interventions in humanitarian crises in LMICs of the MENA region, and will offer additional evidence in support of funding, future research, and policy decisions. We hope to ensure open access to this research to ensure availability to necessary parties through publication in a peer-reviewed journal and conference presentations. In addition, we plan to request that EvidenceAid include a summary of our finished paper to ensure that humanitarian actors have access to the latest evidence [26].

## Conclusion

This scoping review seeks to systematically identify, describe, and assess cancer treatment interventions available to forcibly displaced populations living with cancer in LMIC humanitarian settings across the MENA region. By mapping existing interventions and synthesizing available evidence, this review will address a significant gap in the literature. While humanitarian responses have largely focused on communicable diseases and immediate trauma care, non-communicable diseases such as cancer remain a critical gap, underrepresented in both research and practice.

This study will contribute to the field by offering the first comprehensive overview of cancer interventions in humanitarian contexts within the MENA, providing insights into current practices, challenges, and successes. The review aims to identify key gaps in intervention types, delivery mechanisms, and existing research focuses, thus laying the groundwork for future studies and humanitarian responses supporting forcibly displaced people living with cancer. Ultimately, this review aims to inform policy, guide evidence-based program development, and support the integration of sustainable cancer care into humanitarian health responses.

## Supporting information

S1 Appendix Search strategy for PubMed (National Library of Medicine). (DOCX)

S2 Appendix Data extraction tool. (DOCX)

S3 Appendix PRISMA-P (Preferred Reporting Items for Systematic review and Meta-Analysis Protocols) checklist. (TIF)

## Abbreviations

ACLED: Armed conflict location and event data project
CINAHL: Cumulative index to nursing and allied health literature
GDP: Gross domestic product
LMIC: Low- and middle-income countries
MeSH: Medical subject headings
MENA: Middle East and North Africa
NCD: Non-communicable disease
OCHA: Office for the coordination of humanitarian affairs
OSCE: Organizations for security and co-operation in Europe
PRISMA-ScR: Preferred reporting items for systematic reviews and meta-analyses extension for scoping reviews
UNICEF: United Nations children’s fund
UNHCR: United Nations high commissioner for refugees
WHO: World health organization
WHO EMR: World Health Organization Eastern Mediterranean Region.
