# Supplementary material for: Cancer care interventions for forcibly displaced populations in low- and middle-income countries of the Middle East and North African region affected by humanitarian crises: Protocol for a scoping review
Source: PLoS One. 2025 Aug 18;20(8):e0327946. doi: 10.1371/journal.pone.0327946 (PMC12360602; doi:10.1371/journal.pone.0327946)
Supplement: S1 Appendix — (DOCX) [file pone.0327946.s001.docx]

# S1 Appendix: Search strategy for PubMed (National Library of Medicine)

(neoplasms[mh] OR neoplasm*[tw] OR cancer*[tw]) AND (“Warfare and Armed Conflicts”[mh] OR "armed conflict*"[tw] OR war[tw] OR wars[tw] OR conflict[tw] OR conflicts[tw] OR warfare*[tw] OR humanitarian*[tw] OR refugees[mh] OR refugee*[tw] OR “Transients and Migrants”[mh] OR migrant*[tw] OR "Fragile and Conflict Affected Settings"[tw] OR “Emigrants and Immigrants”[mh] OR Emigrant*[tw] OR Immigrant*[tw] OR “asylum seek*”[tw] OR (forcib*[tw] AND displac*[tw]) OR (internal*[tw] AND displac*[tw]) OR (IDP[tiab] AND displac*[tw]) OR (IDPs[tiab] AND displaced[tw]) OR “refugee camp*”[tw]) AND (Djibouti*[tw] OR Egypt*[tw] OR Jordan[tw] OR Jordanian*[tw] OR Lebanon[tw] OR Lebanese[tw] OR Mauritania*[tw] OR Somalia*[tw] OR "Palestin*"[tw] OR "west bank"[tiab] OR Gaza*[tiab] OR Sudan*[tw] OR Sudan*[tw] OR Syria*[tw] OR Yemen*[tw] OR "middle east*"[tw] OR “Eastern Mediterranean”[tw] OR "North Africa*"[tw] OR "West Africa*"[tw] OR "East Africa*"[tw] OR “Africa, Western”[mh] OR “Africa, Eastern”[mh] OR “Africa, Northern”[mh] OR “North Africa*”[tw] OR Middle East[mh] OR “MENA”[tw] OR "Middle East and North Africa"[tw])
