# Supplementary material for: Cancer care interventions for forcibly displaced populations in low- and middle-income countries of the Middle East and North African region affected by humanitarian crises: Protocol for a scoping review
Source: PLoS One. 2025 Aug 18;20(8):e0327946. doi: 10.1371/journal.pone.0327946 (PMC12360602; doi:10.1371/journal.pone.0327946)
Supplement: S2 Appendix — (DOCX) [file pone.0327946.s002.docx]

# S2 Appendix: Data extraction tool

| Study details | | |
| --- | --- | --- |
| Digital Object Identifier (DOI) |  |  |
| Title |  |  |
| Authors |  |  |
| Official publication date |  |  |
| Full citation: journal, volume number, issue, pages |  |  |
| Research objectives or study aims as reported in full text article (copy-paste) |  |  |
| **Evidence source characteristics** (entered by the coder)^1^ | | |
| Participants: age, sex, quantity |  |  |
| Type of participants: refugees, asylum seekers, etc. |  |  |
| Country/geographic region of study |  |  |
| Country/geographic region of origin of study participants |  |  |
| Study setting: refugee camp, urban clinic, hospital, etc. |  |  |
| Year(s) of study |  |  |
| Context of humanitarian crisis: environmental/natural event or complex emergency |  |  |
| Type of natural/environmental event: earthquake, etc. |  |  |
| Type of complex emergency: civil war, political crisis, economic crisis, etc. |  |  |
| Type of evidence source: peer-reviewed, non-peer-reviewed, gray literature |  |  |
| Type of study: mixed methods, quantitative, qualitative |  |  |
| Type of data collection: survey, focus group discussion, etc. |  |  |
| Results extracted from the source of evidence | | |
| Type of intervention for cancer for the study population |  |  |
| Timeframe of intervention: ongoing, stipulated number of treatments or timeline, etc. |  |  |
| Healthcare delivery setting of intervention: primary health center, hospital facility, mobile clinic, etc. |  |  |
| Measure(s) of cancer intervention |  |  |
| Outcome(s) reported for cancer intervention(s) |  |  |
| Type of medical treatment for cancer for the study population |  |  |
| Funding source of intervention(s) |  |  |
| Similarities/differences of intervention compared to the country’s nationals/citizens |  |  |
| Type of healthcare resources available pre-/post-intervention to the population |  |  |
| Future recommendations of cancer care by author(s) |  |  |
| Funding source(s) of publication |  |  |
| Were the authors all based in a HIC? LMIC? What about the country where the study took place? |  |  |
| Is the paper free to read on the journal website? Elsewhere? |  |  |
| Language of initial publication |  |  |
| Any additional notes |  |  |

^1^ Adapted from: Kumar N, Janmohamed K, Nyanchoka M, Malla M, Baldacchino A, Nafeh F, et al. Substance use and substance use disorder, in relation to COVID-19: protocol for a scoping review. Syst Rev. 2021;10(1):48
