# Supplementary figures and images for: Cancer care interventions for forcibly displaced populations in low- and middle-income countries of the Middle East and North African region affected by humanitarian crises: Protocol for a scoping review
Source: PLoS One. 2025 Aug 18;20(8):e0327946. doi: 10.1371/journal.pone.0327946 (PMC12360602; doi:10.1371/journal.pone.0327946)

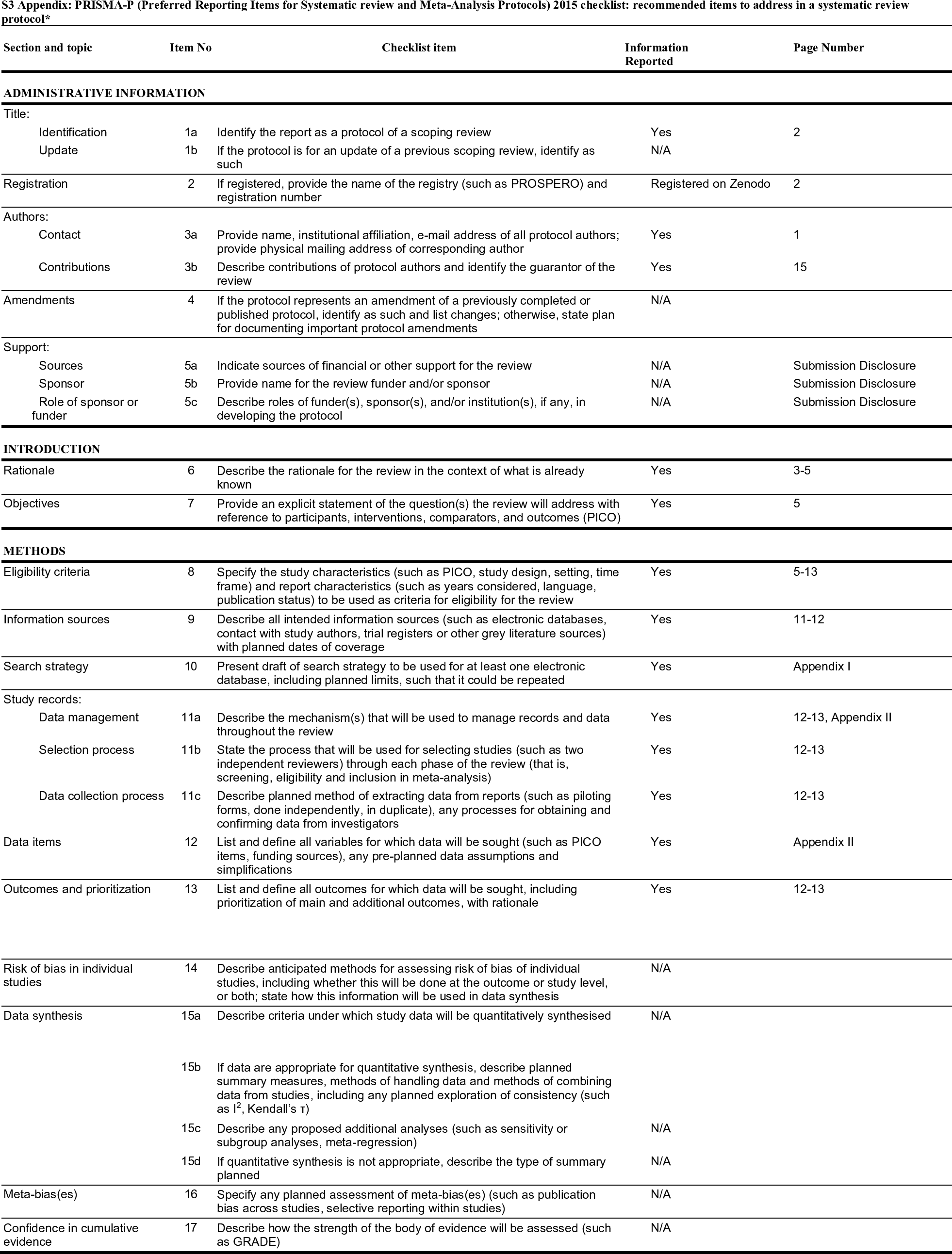

Supplement: S3 Appendix — (TIF) [file pone.0327946.s003.tif]
